# Corticosteroids with low glucocorticoid activity as a potential therapeutic strategy for post‐COVID‐19 myalgic encephalomyelitis/chronic fatigue syndrome in patients with bipolar affective disorder: A case report

**DOI:** 10.1002/pcn5.70222

**Published:** 2025-10-12

**Authors:** Kan Nakajima, Nobutaka Ayani, Teruyuki Matsuoka, Kenya Kasahara, Yoshiyuki Nakajima, Haruki Ikawa, Riki Kitaoka, Tatsuhiko Akimoto, Jin Narumoto

**Affiliations:** ^1^ Department of Psychiatry NHO Maizuru Medical Center Maizuru‐shi Kyoto Japan; ^2^ Department of Psychiatry Graduate School of Medical Science Kyoto Prefectural University of Medicine Kamigyo‐ku Kyoto Japan; ^3^ Department of Endocrinology and Metabolism Graduate School of Medical Science Kyoto Prefectural University of Medicine Kamigyo‐ku Kyoto Japan; ^4^ Department of Psychiatry Higashikouri Hospital Hirakata‐shi Osaka Japan; ^5^ Department of Psychiatry Kitayama Hospital Sakyo‐ku Kyoto Japan

**Keywords:** antipsychotics, bipolar affective disorder, myalgic encephalomyelitis/chronic fatigue syndrome, post‐COVID, steroid switch

## Abstract

**Background:**

The COVID‐19 pandemic has led to an increase in post‐acute sequelae, including myalgic encephalomyelitis/chronic fatigue syndrome (ME/CFS), potentially mediated by dysfunction of the hypothalamic–pituitary–adrenal (HPA) axis. Corticosteroids are occasionally administered to ameliorate fatigue symptoms in ME/CFS; however, their psychiatric adverse effects, particularly in individuals with preexisting mood disorders, necessitate careful consideration.

**Case Presentation:**

We report the case of a 32‐year‐old woman with bipolar disorder who developed ME/CFS following COVID‐19 infection. Initial corticosteroid therapy with betamethasone and prednisolone, agents with potent glucocorticoid receptor (GR) activity, resulted in a manic episode with psychotic features, necessitating psychiatric hospitalization. Although mood stabilization was achieved with olanzapine and valproate, corticosteroid withdrawal subsequently led to metabolic alkalosis and hypoxemia, secondary to hypothalamic hypoadrenalism. Following a comprehensive endocrinological assessment, physiological replacement therapy with hydrocortisone, characterized by relatively higher mineralocorticoid receptor (MR) activity and lower GR potency, was initiated, resulting in the resolution of physical symptoms without destabilization of psychiatric status.

**Conclusion:**

The clinical course suggests that GR‐dominant corticosteroids may exacerbate psychiatric instability in patients with mood disorders. Simultaneously, MR‐favoring agents, such as hydrocortisone, may offer a safer therapeutic alternative for managing HPA axis dysfunction. This case underscores the critical role of receptor selectivity in corticosteroid therapy, particularly in patients with comorbid psychiatric conditions, and highlights the necessity for individualized treatment strategies that integrate both endocrine and neuropsychiatric considerations.

## BACKGROUND

The global coronavirus disease 2019 (COVID‐19) epidemic has led to a range of post‐acute sequelae, collectively referred to as post‐COVID, in addition to the effects of the infection. Post‐COVID has a variety of symptoms, one of which is myalgic encephalomyelitis/chronic fatigue syndrome (ME/CFS), characterized by chronic fatigue that does not improve with adequate rest. One of the key endocrine systems affected by severe acute respiratory syndrome coronavirus 2 (SARS‐CoV‐2) is the hypothalamic–pituitary–adrenal (HPA) axis.^1^ Dysfunction of the HPA axis has been observed in patients with ME/CFS,^2^ with evidence suggesting that ME/CFS following COVID‐19 may also result from dysfunction of the HPA axis.^3^

Graded exercise therapy and cognitive behavioral therapy have been shown to alleviate ME/CFS symptoms, while corticosteroids have demonstrated partial efficacy in improving symptoms.^4^ However, corticosteroid treatment is associated with the risk of exacerbating psychiatric symptoms, likely due to excessive glucocorticoid (GC) effects that suppress endogenous mineralocorticoid (MC) activity.^5^

In this case report, we describe a patient with bipolar disorder who developed ME/CFS following COVID‐19 infection. The patient experienced worsening mania after treatment with betamethasone (BMS) and prednisolone (PSL), both of which have potent GC receptor (GR) effects, and required hospitalization. However, her physical symptoms improved without exacerbation of her psychiatric condition when hydrocortisone, which has relatively strong MC receptor (MR) effects, was introduced.

Attention to the balance between GC and MC activity may help mitigate the risk of psychiatric deterioration. In addition, even during the worsening of psychiatric symptoms associated with steroid use, attention to the balance between GR and MR effects may minimize the use of antipsychotics and other drugs and reduce the risk of adverse effects.

## CASE PRESENTATION

The patient is a 32‐year‐old Japanese female. When she was in her second year of high school (16 years old), she experienced a depressed mood and poor appetite, leading to low body weight and requiring hospitalization for approximately 2 months. During the same period, she also took an overdose of prescribed medication. After graduating from high school, she attended a nursing school, which she dropped out of due to severe insomnia, and began receiving outpatient treatment at a psychiatric clinic. She subsequently changed several jobs. She married in her 20s, gave birth to two children, and later divorced. She then lived with her partner and two children. In 2018, she was hospitalized due to depressive stupor and diagnosed with depression. In 2021, she presented with hypomanic symptoms, leading to a revised diagnosis of bipolar disorder. Initially, sodium valproate (VPA) was prescribed, which was discontinued due to her desire to become pregnant, and the medication was changed to lamotrigine. In May 2022, she contracted COVID‐19, which was followed by persistent fatigue and malaise. A rash was observed on her hands and feet, which was considered unlikely to be a drug reaction, although lamotrigine was discontinued at her request. In August 2023, she was hospitalized for worsening depressive symptoms and started quetiapine; however, this was discontinued due to liver dysfunction, and olanzapine (OLZ) 5 mg/day was initiated, which improved her symptoms. After discharge, she continued the same dose of OLZ, and her mental status remained stable despite persistent fatigue, leading to a diagnosis of post‐COVID syndrome.

Treatment was initiated with oral BMS 0.5 mg and PSL 2.5 mg; however, the effect was limited, and weekly injections of PSL 40 mg were added. This led to the onset of severe mania with delusions of grandeur, such as stating “I am close to God,” and auditory hallucinations, such as stating “An old man's voice is too loud” around March 2024. In April 2024, her mother took her to our hospital because she was having difficulty living at home, and she was admitted on the same day.

**Figure 1:**
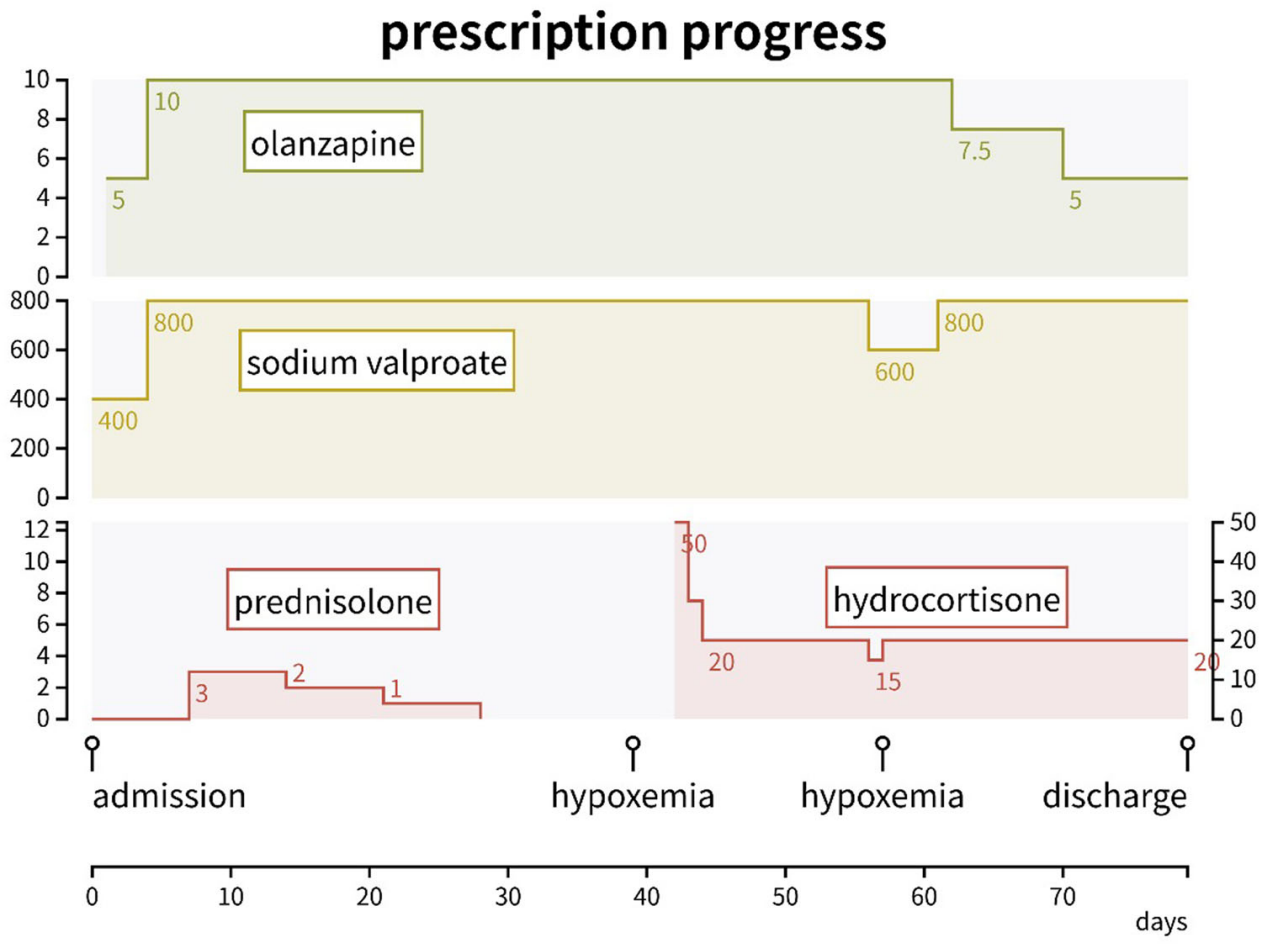
Medication and clinical course during patient hospitalization.

Upon admission, she exhibited marked mood elevation and aggression; therefore, she was secluded and prohibited from meeting with others. She continued to refuse medication; therefore, treatment was administered via intramuscular injection of OLZ. After she was able to take oral medication, she declined to take OLZ due to the risk of weight gain. Since she no longer desired to have children at that time, we prescribed aripiprazole (APZ) and VPA, discontinued BMS, and continued PSL. However, APZ caused akathisia, and the alternative asenapine was difficult to take due to its bitter taste. Ultimately, OLZ (10 mg) was used as the main drug. Under the administration of OLZ 10 mg and VPA 800 mg, although singing, delusions of grandeur, and auditory hallucinations remained, her rage disappeared, and the seclusion and prohibition of meeting with others were lifted on the 26th day of hospitalization. PSL, which may exacerbate psychiatric symptoms, was carefully reduced by 1 mg per week. Ten days after PSL discontinuation, she suddenly developed hypoxemia with SpO_2_ 90%. A search for the cause was conducted in collaboration with the emergency medicine department, which found metabolic alkalosis and determined that compensatory hypoventilation was the cause of hypoxemia. Endocrinologic testing (triple bolus test), including evaluation of anterior pituitary function, revealed hypothalamic hypoadrenalism as the cause of her symptoms. Therefore, hydrocortisone 50 mg/day was introduced, and the patient was followed up with close attention to her psychiatric symptoms, and her physical symptoms improved without worsening of her mental status. Hydrocortisone was then tapered off, and when the dose was reduced to 15 mg, hypoxemia recurred. Therefore, 20 mg was continued, and no relapse of manic episodes was observed thereafter. OLZ was tapered to 5 mg for weight gain, and no worsening of psychiatric symptoms was observed; she was discharged in June 2024 (Figure 1).

## DISCUSSION

Corticosteroids are known to exacerbate psychiatric symptoms, with patients with bipolar disorder particularly vulnerable to manic episodes following corticosteroid use.^6^ The mechanism underlying corticosteroid‐induced psychiatric exacerbation likely involves the depletion of endogenous MR ligands due to the administration of steroids with strong GR effects.^5^ Studies have shown that psychiatric symptoms improve with physiological doses of MR ligands.^7^ Cases of delirium and auditory hallucinations have been reported following a switch from PSL (which has both GR and MR effects) to BMS (which predominantly has GR effects), with improvement upon switching back to PSL.^8^, ^9^

We hypothesized the following sequence of events. First, COVID‐19 led to hypothalamic dysfunction, causing hypothalamic hypoadrenalism and contributing to ME/CFS onset. Next, PSL and BMS were used to treat ME/CFS, improving physical symptoms but worsening psychiatric symptoms of bipolar disorder. Despite a gradual reduction in PSL, the patient still experienced severe physical symptoms due to hypothalamic hypoadrenalism.

Discontinuation of corticosteroids remains the first‐line treatment for psychiatric exacerbations caused by corticosteroid therapy. However, when discontinuing corticosteroids is challenging due to the treatment of physical illness, antipsychotics or mood stabilizers are used to control mental status. The patient's psychiatric symptoms were stabilized with OLZ and VPA; however, reducing the corticosteroid dose sufficiently to treat her physical illness remained difficult, hindering psychiatric control. Switching to hydrocortisone, which has stronger MR effects than PSL,^10^ improved psychiatric symptoms while stabilizing her physical condition. Treatment with GR ligands alone carries a high risk of steroid‐induced psychiatric symptoms.^5^ In this case, hydrocortisone, with its weak GR effect, combined with psychotropic drugs, likely contributed to preventing recurrent manic symptoms.

Blind administration of corticosteroids in patients with ME/CFS is not advisable; however, corticosteroid replacement therapy may be warranted in cases where patients have conditions such as hypothalamic hypoadrenalism. Therefore, evaluating hypothalamic function may contribute to improving the quality of ME/CFS treatment. In particular, in patients with mental disorders, corticosteroid administration may exacerbate mental instability; therefore, we believe that conducting hypothalamic function tests is meaningful to evaluate the appropriateness of corticosteroid administration. In psychiatric patients with concurrent adrenal insufficiency, corticosteroid therapy should be carefully administered in close collaboration between endocrinology and psychiatry, with strict physical management and evaluation of psychiatric symptoms. Furthermore, attention to the balance between GC and MC activity may help mitigate the risk of psychiatric deterioration. In addition, even during the worsening of psychiatric symptoms associated with steroid use, attention to the balance between GR and MR effects may minimize the use of antipsychotics and other drugs and reduce the risk of adverse effects.

## CONCLUSION

Corticosteroid therapy may benefit patients with ME/CFS with hypothalamic hypoadrenalism. For those with mental disorders like bipolar disorder, careful selection of corticosteroids based on GR and MR effects may help prevent psychiatric exacerbation.

## AUTHOR CONTRIBUTIONS

**Kan Nakajima**: Conceptualization; data curation; writing—original draft. **Nobutaka Ayani**: Conceptualization; data curation; supervision; writing—review and editing. **Teruyuki Matsuoka**: Supervision; writing—review and editing. **Kenya Kasahara**: Data curation; writing—review and editing. **Yoshiyuki Nakajima**: Data curation; writing—review and editing. **Haruki Ikawa**: Data curation; writing—review and editing. **Riki Kitaoka**: Data curation; writing—review and editing. **Tatsuhiko Akimoto**: Data curation; writing—review and editing. **Jin Narumoto**: Conceptualization; supervision; writing—review and editing.

## CONFLICT OF INTEREST STATEMENT

The authors declare no conflicts of interest.

## ETHICS APPROVAL STATEMENT

All procedures involving the human patient were performed in accordance with the Declaration of Helsinki. Consent was obtained from the family instead of ethics committee approval because this is a case report.

## CLINICAL TRIAL REGISTRATION

N/A.

## PATIENT CONSENT STATEMENT

Written informed consent was obtained from the patient.

## Data Availability

The data that support the findings of this study are available on request from the corresponding author. The data are not publicly available due to privacy or ethical restrictions.
